# Computer-assisted total hip arthroplasty reduces early complications based on Japanese nationwide medical claims data

**DOI:** 10.1007/s00402-025-06116-z

**Published:** 2025-11-12

**Authors:** Hidetatsu Tanaka, Kunio Tarasawa, Yu Mori, Hiroaki Kurishima, Hiroki Kawamata, Kiyohide Fushimi, Kenji Fujimori, Toshimi Aizawa

**Affiliations:** 1 Department of Orthopaedic Surgery, Tohoku University Graduate School of Medicine, Sendai, Japan; 2 Department of Health Administration and Policy, Tohoku University Graduate School of Medicine, Sendai, Japan; 3 Department of Orthopaedic Surgery, Japanese Redcross Sendai Hospital, Sendai, Japan; 4 Department of Health Policy and Informatics, Institute of Science Tokyo, Tokyo, Japan

## Abstract

Introduction: Computer-assisted (CA) surgery is increasingly adopted in total hip arthroplasty (THA) to enhance implant positioning accuracy. However, robust evidence regarding its impact on postoperative complications compared with manual THA (M-THA) remains limited. This study evaluated the association between CA-THA and early postoperative complications using a large Japanese database. Materials and Methods: We analyzed 336,624 THA cases recorded in the Japanese Diagnosis Procedure Combination (DPC) database between December 2011 and March 2023. Of these, 242,067 underwent M-THA and 94,557 underwent CA-THA. One-to-one propensity score matching was performed to adjust for age, sex, body mass index, comorbidities, and diagnosis. Outcomes included surgical complications, medical complications, and in-hospital mortality. Results: After matching, 93,887 patient pairs were analyzed. Compared with M-THA, CA-THA was associated with lower odds of dislocation (OR 0.667, 95% CI 0.556–0.786, *p* < 0.001), infection (OR 0.763, 95% CI 0.687–0.848, *p* < 0.001), and re-operation (OR 0.822, 95% CI 0.732–0.922, *p* < 0.001), but higher odds of periprosthetic fracture (OR 1.301, 95% CI 1.118–1.514, *p* < 0.001). No significant differences were found in medical complications or mortality. Conclusions: In this nationwide cohort, CA-THA was associated with reduced risks of dislocation, infection, and reoperation, but an increased risk of periprosthetic fracture compared with M-THA. Further research should clarify optimal indications and refine patient selection criteria for CA-THA. In this nationwide cohort, CA-THA was associated with reduced risks of dislocation, infection, and reoperation, but an increased risk of periprosthetic fracture compared with M-THA. Further research should clarify optimal indications and refine patient selection criteria for CA-THA.

## Introduction

 Total hip arthroplasty (THA) is a common orthopedic procedure used to treat patients with symptomatic hip arthritis and has been recognized as one of the most successful surgeries of the past century [26]. As is well known, prosthetic hip dislocation is a relatively rare but serious complication and one of the most common indications for revision total hip arthroplasty [4, 6, 15]. Several studies have identified malpositioning of the acetabular component in THA as a major risk factor for dislocation [1, 5, 8, 13, 21, 28, 29, 33]. To enhance the accuracy of implant positioning, various technologies have been developed and are now in use, including computer-assisted navigation systems, robotic arm-assisted surgery, and simplified navigation systems [10, 11, 23, 34, 35, 43]. Computer-assisted systems are also used in osteotomy and hip arthroscopic surgery, with an expanding range of clinical uses [18].

Registry data from Australia showed that computer‑navigated THA had a lower 10‑year revision rate for dislocation [2]. In contrast, a large database analysis reported higher rates of periprosthetic fracture and revision with computer‑navigated primary THA [30]. Although computer-assisted THA can improve implant positioning, evidence on whether it reduces complications or offers clear advantages over manual THA remains conflicting, and no consensus has been reached [2, 3, 11, 16, 25, 30, 38, 40, 41]. Several large-scale comparative studies reported that the use of computer-assisted navigation or robotic arm-assisted THA reduces the risk of dislocation [11, 19, 37], while others report no significant effect [30, 36]. Given the multifactorial nature of complications, comparisons using controlled backgrounds are preferable. Howell et al. compared robotic- with manual THA using propensity score matching for age, sex, and BMI, and found fewer readmissions for dislocation and infections in the robotic group [17]. Conversely, Gregory J. Kirchner reported that, after adjusting for background factors, the risk of serious complications in robotic-assisted THA patients was similar to that in the conventional THA group [22]. While some studies suggest that computer-navigated THA results in lower complication and readmission rates compared to traditional THA, no significant difference has been found in the rate of revision surgery within the first 90 days after adjusting for confounding variables [14].

Use of robotic‑assisted THA has grown in the United States [11, 42], and navigation, robotics, and simplified systems are increasingly used in Japan. Most complication studies originate from the United States, and large Japanese datasets are scarce. We therefore evaluated whether computer‑assisted THA reduces early postoperative complications compared with manual THA in Japan. Using the Diagnosis Procedure Combination (DPC) database, we analyzed (1) hip and surgical complications, (2) medical complications, and (3) in‑hospital mortality after balancing patient characteristics.

## Materials and methods

This study was conducted using data from the Japanese DPC database following the ethical standards of the Declaration of Helsinki. It was approved by the Institute of Science Tokyo (approval No. M2000-788) and Tohoku University Graduate School of Medicine (approval No. 2021-1-1082 and 2024-1-1026).

### Study design

**Fig. 1:**
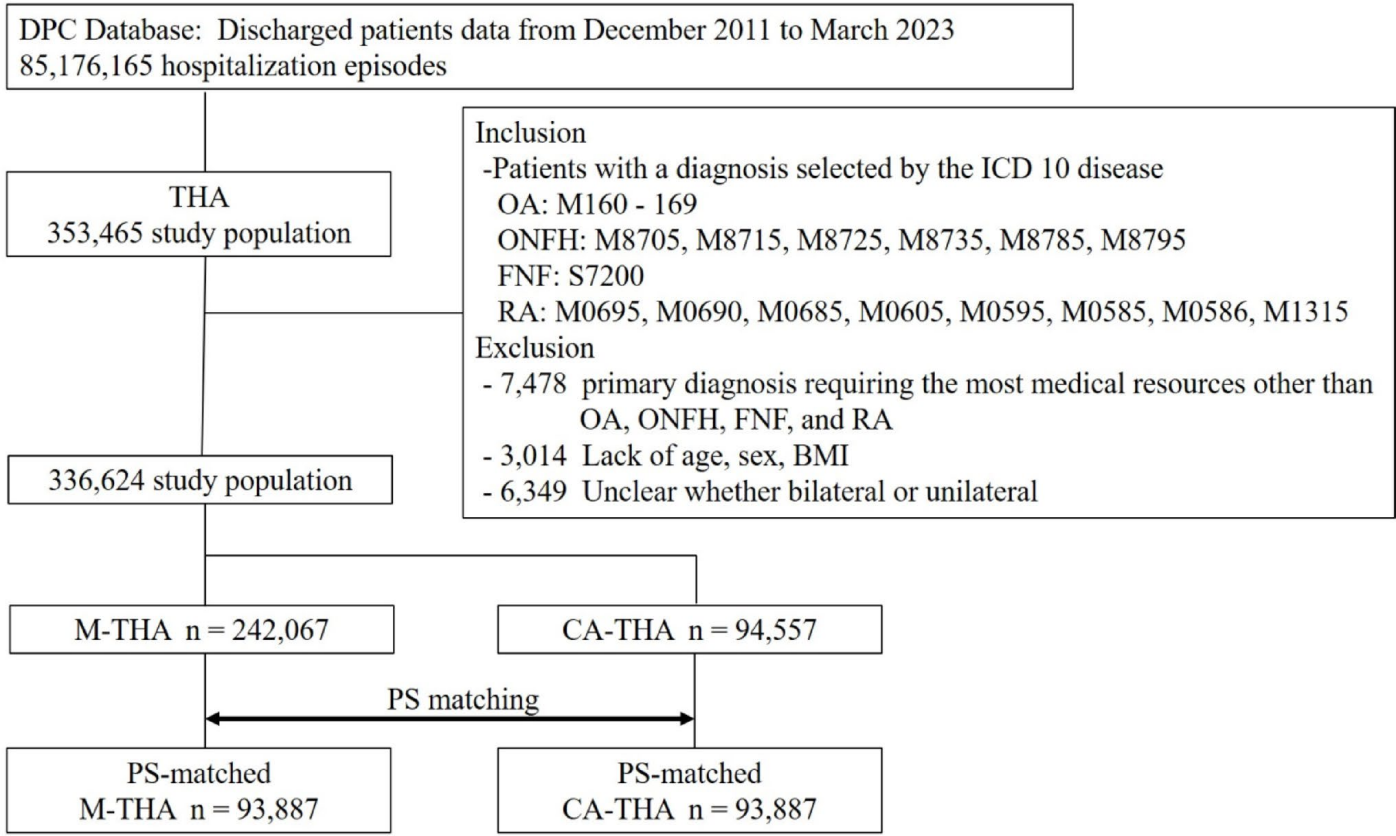
Study flow chart

The DPC data used in this study included approximately 1100 hospitals, covering roughly 70% of all annual hospitalization episodes in Japan, thereby reflecting the country’s clinical practices. The anonymized dataset contains patient age, sex, diagnoses coded according to the International Classification of Diseases, Tenth Revision (ICD-10) [47], hospital admission and discharge dates, discharge status, medications, and procedures, including surgeries and prescribed drugs. Additionally, admission diagnoses, pre-existing comorbidities at admission, and post-admission complications during hospitalization are recorded separately. The Japanese National Administrative DPC Reimbursement System retrospectively reviewed the database. The inclusion criteria for the study were as follows: (1) THA: receipt code for the surgical procedure of 150,050,410, (2) primary diagnosis requiring the most medical resources: Osteoarthritis of the hip (ICD-10: M160-M169), (3) osteonecrosis of the femoral head (ICD-10: M8705, M8715, M8725, M8735, M8785, M8795), (4) femoral neck fracture (ICD-10: S7200), (5) Rheumatoid arthritis (ICD-10: M0695, M0690, M0685, M0605, M0595, M0585, M0586, M1315). The exclusion criteria for the study were as follows: (1) primary diagnosis requiring the most medical resources other than Osteoarthritis of the hip, osteonecrosis of the femoral head, femoral neck fracture, and Rheumatoid arthritis, (2) lack of data such as age, sex, and BMI, (3) unclear whether bilateral or unilateral. Patients who underwent THA between December 2011 and March 2023 were included in the DPC database, resulting in a total sample of 336,624 patients. The presence of computer-assisted surgery was determined based on the application of imaging and other surgical support surcharges (K-939 in medical treatment fee points). Of these, 242,067 patients underwent manual THA (M-THA), and 94,557 underwent computer-assisted THA (CA-THA). A flowchart of the study is provided in Fig. 1.

## Data selection

A one-to-one propensity score (PS) matching was performed to compare M-THA and CA-THA groups. The covariates used for confounding adjustment included age, sex, body mass index (BMI), surgical side, diagnosis for THA, and Charlson Comorbidity Index (CCI). C-statistics were used to assess the discriminative power of the models. Propensity score estimates were used to perform nearest-neighbor matching without replacement, with calipers set at 0.2 times the standard deviation of the PS estimate. This process resulted in matched pairs, establishing propensity score-matched control and treatment groups for further analysis.

## Outcomes

Assessed outcomes included postoperative dislocation, infection, periprosthetic fracture, nerve palsy including sciatic and femoral nerve, wound dehiscence, and reoperation as surgical complications. Furthermore, the development of hospital-acquired pneumonia, deep vein thrombosis (DVT), pulmonary embolism (PE), cardiac event, cerebrovascular event, acute renal failure, and mortality during hospitalization as medical complications were investigated. These complications were compared between M-THA and CA-THA cohorts.

## Data analyses

All data are presented as means ± standard deviations (SDs). Significant differences between the two groups were evaluated using the χ² test and Student’s t-test for each parameter. The Kolmogorov-Smirnov test was used to assess the normality of data distribution. Univariate logistic regression analyses were performed to assess the association between the use of CA-THA and the occurrence of the following outcomes: dislocation, infection, periprosthetic fracture, nerve palsy, wound dehiscence, reoperation, pneumonia, DVT, PE, cardiac events, cerebrovascular events, acute renal failure, and in-hospital mortality. Multivariate logistic regression analyses were conducted following the univariate analyses to adjust for potential confounders. The P-value criterion for including variables used in univariate analyses in multivariate analyses was *P* < 0.25 [7]. All statistical tests were two-tailed, and P-values < 0.001 were considered statistically significant. All analyses were performed using JMP version 18.0 (SAS, Cary, North Carolina, USA).

## Results

**Fig. 2:**
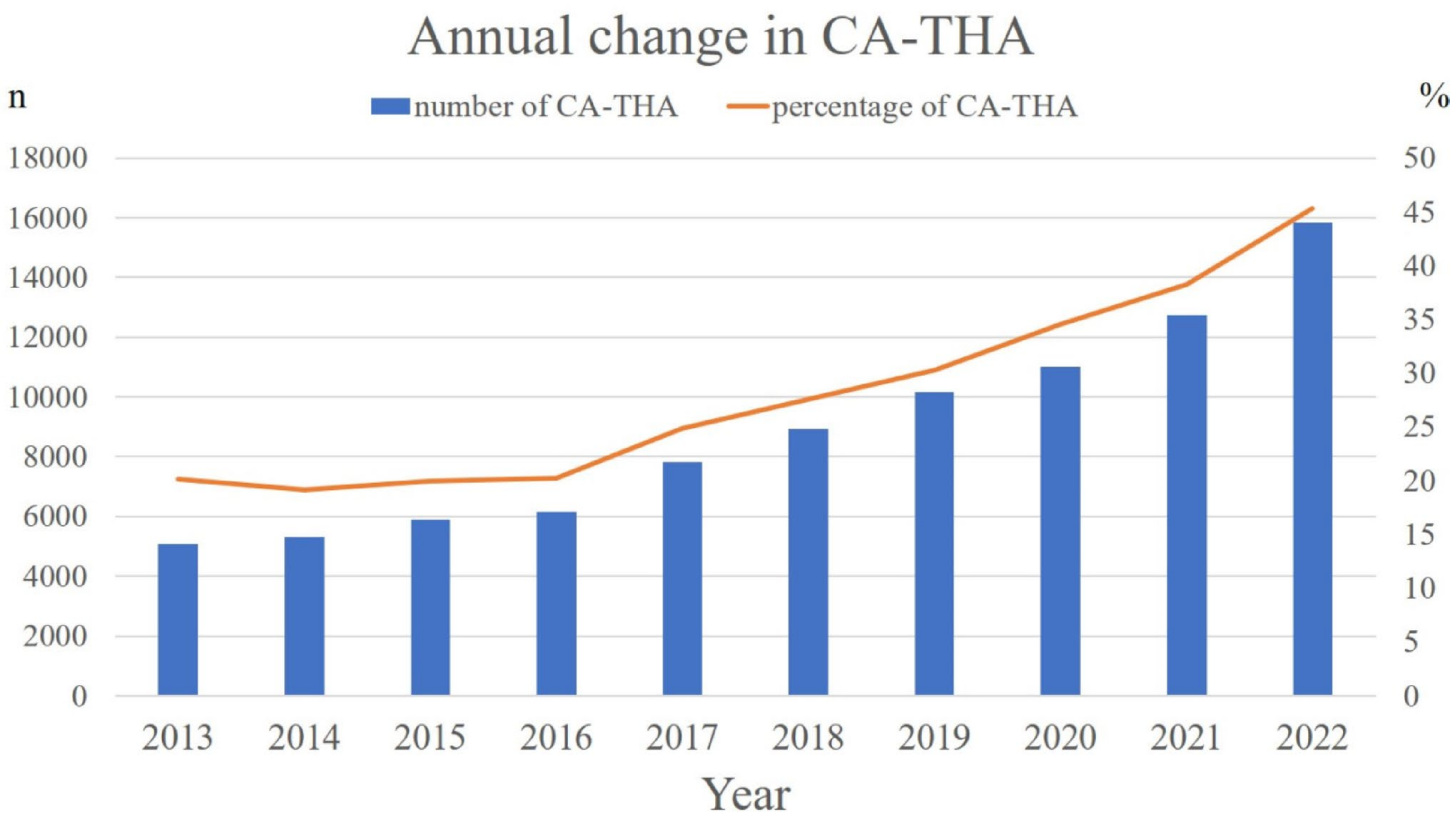
Annual change in CA-THA

After one-to-one PS matching, 93,887 patients were included in both M-THA and CA-THA cohorts. Baseline demographic data were presented in Table 1. The C-statistic was 0.7654, and the standardized mean differences (SMD) for all parameters were < 0.1, indicating a well-balanced dataset. The average length of hospital stay was significantly longer in M-THA patients. The transfusion rate was significantly higher on postoperative day 0 in CA-THA patients but higher on days 1 and 2 in M-THA patients. The osteoporosis treatment rate was higher in CA-THA patients, while the use of anticoagulant and antiplatelet medications was significantly higher in M-THA patients. Additionally, bone cement usage was more common in the M-THA group (Table 1). The adoption of CA-THA has increased over time (Fig. 2).

**Table 1:** Characteristics of patients after propensity score matching

	M-THA	CA-THA	SMD
n	93,887	93,887	
Age	67 ± 10.9	67 ± 11.1	0.005
Gender (%)			
Men	16,037 (17.1)	16,918 (18.0)	0.025
Women	77,850 (82.9)	76,969 (82.0)	
BMI	23.9 ± 4.4	24.0 ± 4.9	0.013
Surgical side (%)			
Unilateral	90,271 (96.2)	90,278 (96.2)	0.000
Bilateral	3616 (3.8)	3609 (3.8)	
Diagnosis for THA (%)			
OA	82,387 (87.8)	82,320 (87.7)	0.001
ONFH	7248 (7.7)	7322 (7.8)	
RA	1035 (1.1)	1078 (1.1)	
FNF	3217 (3.4)	3167 (3.4)	
CCI	0.48 ± 0.85	0.46 ± 0.81	0.023

			*p*-value
Length of hospital stay	27.4 ± 16.7	24.2 ± 13.3	< 0.001※
Transfusion (%)			
Day 0	46,111 (49.1)	51,591 (55.0)	< 0.001
Day 1	22,397 (23.9)	17,995 (19.2)	< 0.001
Day 2	3468 (3.7)	2654 (2.8)	< 0.001
Medications (%)			
Osteoporosis treatment	5078 (5.4)	6476 (6.9)	< 0.001
Anticoagulate agent	70,713 (75.3)	68,985 (73.5)	< 0.001
Antiplatelet agent	7135 (7.6)	6451 (6.9)	< 0.001
Use of bone cement (%)	18,862 (20.1)	10,421 (11.1)	< 0.001

Age, BMI, and Length of hospital stay are shown as mean ± standard deviation

*OA* means osteoarthritis, *ONFH* means osteonecrosis of femora head, *RA* means rheumatoid arthritis, *FNF* means femoral neck fracture, *CCI* means Charlson comorbidity index

*P*-values of < 0.001 are considered significant by the Student t-test andχ2 test

※ Student-t test

The associations between CA-THA and the occurrence of surgical complications were summarized in Table 2. In CA-THA cohort, the risk of postoperative dislocation, infection, and reoperation during hospitalization was significantly lower, with the following odds ratios (OR): dislocation: OR = 0.667 (95% Confidence Interval [CI] 0.556–0.786), infection: OR = 0.763 (95% CI 0.687–0.848), and reoperation: OR = 0.822 (95% CI 0.732–0.922). The risk of periprosthetic fracture was significantly higher, with an OR of 1.301 (95% CI 1.118–1.514). Conversely, the risks of nerve palsy and wound dehiscence were not significant.

The results of the multivariate logistic regression analysis assessing the association between dislocation and factors such as age, sex, BMI, and CA-THA are shown in Table 3. CA-THA was associated with a significantly lower risk of dislocation, with an OR of 0.557. An increase in age by one year with an OR of 1.010, male with an OR of 1.610, and an increase in CCI by one with an OR of 1.331 were significantly associated with dislocation risk.

**Table 2:** Association between CA-THA and surgical complications

Complications	Total (n)		Univariate analysis			Multivariable analysis		
		OR	95% CI	*P-*value	OR	95% CI	χ2 statics	*P*-value
Dislocation	1,120	0.554	0.490–0.626	< 0.001	0.667	0.566–0.786	23.77	< 0.001
Infection	1,431	0.741	0.667–0.824	< 0.001	0.763	0.687–0.848	25.07	< 0.001
Periprosthetic fracture	776	1.168	1.014–1.346	0.034	1.301	1.118–1.514	11.65	< 0.001
Nerve palsy	122	0.877	0.614–1.252	0.526	–	–	–	–
Wound dehiscence	208	1.000	0.762–1.313	1	–	–	–	–
Reoperation	2,400	0.703	0.647–0.762	< 0.001	0.822	0.732–0.922	11.25	< 0.001

**Table 3:** Multivariate logistic analysis of risk factors for surgical complications

Dislocation	Variable	OR	95% CI	χ2 statics	*P*-value
	Age	1.010	1.004–1.015	12.13	< 0.001
	Gender (Men)	1.610	1.403–1.847	42.92	< 0.001
	BMI	1.000	0.991–1.001	1.244	0.265
	Bilateral THA	0.921	0.774–1.525	0.235	0.628
	CCI	1.331	1.264–1.399	100.0	< 0.001
	Computer-assisted surgery	0.557	0.492–0.630	89.78	< 0.001
	Use of bone cement	1.113	0.956–1.295	1.56	0.172
	Osteoporosis treatment	1.445	1.175–1.778	11.06	0.009
Infection	Age	1.003	0.998–1.007	1.529	0.216
	Gender (Men)	1.607	1.424–1.814	55.07	< 0.001
	BMI	1.013	1.008–1.019	21.37	< 0.001
	Bilateral THA	0.881	0.653–1.188	0.714	0.398
	CCI	1.284	1.224–1.345	90.12	< 0.001
	Computer-assisted surgery	0.738	0.664–0.820	32.24	< 0.001
	Osteoporosis treatment	1.245	1.022–1.517	4.460	0.035
Periprosthetic	Age	1.027	1.020–1.034	58.06	< 0.001
Fracture	Gender (Men)	1.254	1.020–1.542	4.863	0.027
	BMI	0.984	0.966–1.001	3.494	0.062
	Bilateral THA	2.230	1.693–2.940	26.29	< 0.001
	CCI	1.185	1.010–1.272	18.49	< 0.001
	Computer-assisted surgery	1.121	0.973–1.293	2.50	0.114
	Use of bone cement	0.510	0.397–0.655	33.22	< 0.001
	Osteoporosis treatment	1.094	0.841–1.422	0.436	0.509
Re-operation	Age	1.006	1.002–1.010	10.08	0.002
	Gender (Men)	1.286	1.164–1.421	23.54	< 0.001
	CCI	1.242	1.194–1.290	104.2	< 0.001
	Computer-assisted surgery	0.703	0.648–0.763	72.25	< 0.001
	Osteoporosis treatment	1.171	1.003–1.368	3.845	0.050

*OR* means odds ratio, *CCI* means Charlson comorbidity index, *CI* means confidence interval

*P*-values of < 0.001 are considered significant by the χ2 test

The outcomes of the multivariate logistic analysis for assessing risk factors for infection are shown in Table 3. For postoperative infection, CA-THA was associated with a significantly lower risk of infection, with an OR of 0.738. Males with an OR of 1.718, an increase in BMI by one with an OR of 1.013, and an increase in CCI by one with an OR of 1.284 significantly elevated the risk.

The results of the multivariate logistic analysis for assessing risk factors for periprosthetic fracture are shown in Table 3. An increase in age by one year with an OR of 1.027, bilateral THAs on the same day with an OR of 2.230, and an increase in CCI by one with an OR of 1.185 were significantly elevated risk factors. The use of bone cement was associated with a lower risk of periprosthetic fracture, with an OR of 0.510. In contrast, sex, BMI, CA-THA, and osteoporosis treatment were not associated with the apparent risk of periprosthetic fracture.

The results from the multivariate logistic regression analyses aimed at identifying risk factors for re-operation during hospitalization are described in Table 3. The CA-THA was at a lower risk of re-operation, with an OR of 0.703. Male sex with an OR of 1.286 and an increase in CCI by one with an OR of 1.242 were demonstrated as significantly elevated risk factors.

The associations between the CA-THA and the development of medical complications were described in Table 4; significant associations were not found in univariate and multivariable analyses.

**Table 4:** Association between CA-THA and medical complications

Complications	Total (n)		Univariate analysis			Multivariable analysis		
		OR	95% CI	*P*-value	OR	95% CI	χ2 statics	*P*-value
Hospital-acquired pneumonia	256	0.910	0.712–1.164	0.492	–	–	–	–
DVT	9,737	0.968	0.930–1.009	0.129	0.970	0.931–1.010	2.224	0.137
PE	328	0.802	0.645–0.997	0.053	0.804	0.647–1.000	3.857	0.050
Cardiac event	28	0.647	0.303–1.381	0.345	–	–	–	–
Cerebrovascular event	353	1.166	0.946–1.438	0.166	1.170	0.949–1.443	2.174	0.140
Acute renal failure	41	0.640	0.342–1.199	0.211	0.642	0.343–1.204	1.956	0.162
Mortality during hospitalization	581	0.787	0.668–0.927	0.005	0.786	0.667–0.927	8.305	0.004

*OR* means odds ratio, *CI* means confidence interval

*P*-values of < 0.001 are considered significant by the χ2 test

## Discussion

In this study, we investigated whether CA-THA lowers the risk of postoperative complications compared to M-THA using the Japanese DPC database. Our analysis revealed that CA-THA was significantly associated with a lower risk of postoperative dislocation, infection, and reoperation compared with M-THA. However, no significant differences were observed in perioperative fractures, nerve palsy, or wound dehiscence incidence. A key strength of this study was the use of a large-scale Japanese database, encompassing approximately 330,000 cases, which allowed for robust statistical analysis and enhanced the reliability of assessing the effectiveness of CA-THA. Additionally, PS matching was employed to minimize the influence of confounding factors, such as age, sex, BMI, surgical side, and disease background, enabling more accurate comparisons between CA-THA and M-THA. Few studies in Japan have utilized large-scale data for such analyses. Our previous research on hip degenerative disease and proximal femur fractures using DPC data, including this study, has contributed to a better understanding of the overall landscape of orthopedic medical care in Japan [31, 32, 44, 45].

The results of this study were consistent with previous reports in many areas, and multivariate analysis suggests that CA-THA may reduce the risk of dislocation and infection. Previous studies had reported a reduction in dislocation rates with the use of CA-THA [11, 17, 37], and this study also confirmed that CA-THA is associated with a reduced risk of postoperative dislocation. It was widely accepted that improving the acetabular cup placement angle is a key factor in reducing dislocation risk. However, some studies have found no significant difference in dislocation rates between CA-THA and manual THA [19, 25, 30]. Possible explanations included the multifactorial nature of dislocations, the occurrence of dislocations even within the Lewinnek safe zone, and the lack of studies demonstrating an actual reduction in hip joint instability [30]. Determining the optimal cup placement and the ideal location of the hip joint center remained a critical challenge. However, we anticipated that research utilizing computer-assisted surgery would eventually yield definitive results. While postoperative dislocation following THA was known to be multifactorial [9, 24, 39], CA-THA may provide an effective and efficient solution for improving THA outcomes.

The CA-THA significantly reduced the risk of postoperative infection, with similar demographic information and mean CCI scores between cohorts. This might be because CA-THA allows for proper positioning of the implant, optimizing surgical procedures, reducing unnecessary tissue damage, and reducing human error. A similar trend had been reported regarding the reduction in infection risk, which might be due to shorter hospital stays and a lack of intraoperative fluoroscopy [17]. However, some studies have suggested that CA-THA may increase the risk of postoperative complications, hypothesizing that the insertion site of the reference marker pin is a contributing factor [3]. The complication rate associated with CA pins in total hip arthroplasty has been reported to be 0.46%, with all complications successfully managed with nonoperative treatment. In cases where infections occurred, oral antibiotics were sufficient for resolution [20]. There was no significant difference in infection rates between CA-THA and manual THA[11; 19], indicating the need for further investigation.

Regarding peri-implant fractures, the unadjusted analysis showed a higher periprosthetic fracture rate in the CA-THA group. However, multivariate analysis revealed that this association was largely explained by patient- and surgery-related factors. Specifically, older age, undergoing bilateral THA on the same day, and higher Charlson Comorbidity Index scores were significant independent predictors of fracture, while CA-THA itself was not a significant factor after adjustment. In addition, bone cement use, more frequent in the M-THA group, was associated with a lower fracture risk, which was consistent with previous reports showing that cemented fixation reduces postoperative fracture incidence [12, 27]. These findings suggested that the initially observed higher fracture rate in CA-THA was influenced by underlying patient characteristics and surgical factors, rather than the computer-assisted technique itself. It was reported that robotic-assisted THA had a higher rate of peri-implant fractures compared to conventional THA [30], but this clinical trial showed no difference between robotic-assisted THA and navigation-assisted THA [37], which was different from previous reports.

The reoperation rate had been reported as not significantly different between CA-THA and manual THA [14]. A study reported the revision rate was approximately 0.4% higher at 30 days and persisted at 90 days as CAS-THA remained 0.5% higher than the conventional THA [30]. They hypothesize that the increase in early revision rate might be partially due to the learning curve induced by implementing CAS-THA [30]. The results of this study showed that CA-THA significantly reduced the risk of reoperation, likely due to a reduced incidence of complications requiring reoperation. However, a reduction in overall complication rates contributed to a decrease in the reoperation rate over time.

Similar to the findings of this study, previous research had reported no significant differences in medical complications between CA-THA and manual THA [11, 19]. However, other studies had suggested that CA-THA was associated with a lower incidence of pneumonia [14], while robotic-assisted THA increased the risk of PE, whereas navigated THA may reduce it [11]. Additionally, CA-THA has been reported to reduce the risk of DVT and PE [30]. Although the impact of CA-THA on medical complications appeared to be minimal, further investigation is necessary to fully interpret these findings. The clinical significance of this study lies in its indication that the introduction of CA-THA might contribute to improved postoperative outcomes. In particular, CA-THA was especially beneficial for elderly patients and those at high risk of dislocation. There had been ongoing discussion regarding the cost-effectiveness of computer-assisted surgery [11]. The implementation of CA-THA presents challenges such as high costs, increased surgical time, and the need for specialized training. Therefore, it was crucial to carefully evaluate its suitability by weighing its benefits against its limitations.

This study had several potential limitations. The study population included only patients who underwent THA in hospitals reporting to the DPC data system, excluding those admitted to non-DPC reporting beds, which account for 30% of all general hospital beds [46]. Additionally, this study lacked detailed information regarding the specific type of computer-assisted technology used, such as navigation systems, robotic arm-assisted systems, or simplified navigation systems [10, 23, 34, 35, 43]. Furthermore, data on surgical approach, implant types, blood test data, bleeding volume, surgeon identifiers, surgical volume, years of experience, surgery time, process of diagnosing complications, and implant positioning were unavailable. Another limitation was that the DPC database only captures complications occurring during hospitalization, preventing an assessment of long-term outcomes after discharge. Therefore, further studies utilizing long-term follow-up data were necessary to determine the long-term efficacy and safety of CA-THA.

## Conclusion

In conclusion, this large-scale study using a Japanese database demonstrated that CA-THA may reduce the risks of postoperative dislocation, infection, and reoperation compared with M-THA. Moving forward, it will be crucial to further clarify the indications for CA-THA and establish guidelines for selecting the optimal surgical approach for each patient.

## Data Availability

No datasets were generated or analysed during the current study.

## References

[1] Abdel MP, von Roth P, Jennings MT, Hanssen AD, Pagnano MW (2016) What safe zone? The vast majority of dislocated THAs are within the Lewinnek safe zone for acetabular component position. Clin Orthop Relat Res 474(2):386–391 26150264 10.1007/s11999-015-4432-5 PMC4709312

[2] Agarwal S, Eckhard L, Walter WL et al (2021) The use of computer navigation in total hip arthroplasty is associated with a reduced rate of revision for dislocation: a study of 6,912 navigated THA procedures from the Australian orthopaedic association National joint replacement registry. J Bone Joint Surg Am 103(20):1900–1905 34143758 10.2106/JBJS.20.00950

[3] Aoude AA, Aldebeyan SA, Nooh A, Weber MH, Tanzer M (2016) Thirty-Day complications of conventional and Computer-Assisted total knee and total hip arthroplasty: analysis of 103,855 patients in the American college of surgeons National surgical quality improvement program database. J Arthroplasty 31(8):1674–1679 26923496 10.1016/j.arth.2016.01.042

[4] Bains SS, Dubin JA, Salib CG et al (2024) The epidemiology of the revision total hip arthroplasty in the United States from 2016 to 2022. Arthropl Today 30:101517 10.1016/j.artd.2024.101517 PMC11550771 39524991

[5] Biedermann R, Tonin A, Krismer M, Rachbauer F, Eibl G, Stockl B (2005) Reducing the risk of dislocation after total hip arthroplasty: the effect of orientation of the acetabular component. J Bone Joint Surg Br 87(6):762–769 15911655 10.1302/0301-620X.87B6.14745

[6] Bozic KJ, Kurtz SM, Lau E, Ong K, Vail TP, Berry DJ (2009) The epidemiology of revision total hip arthroplasty in the United States. J Bone Joint Surg Am 91(1):128–133 19122087 10.2106/JBJS.H.00155

[7] Bursac Z, Gauss CH, Williams DK, Hosmer DW (2008) Purposeful selection of variables in logistic regression. Source Code Biol Med 3:17 19087314 10.1186/1751-0473-3-17 PMC2633005

[8] D’Lima DD, Urquhart AG, Buehler KO, Walker RH, Colwell CW Jr (2000) The effect of the orientation of the acetabular and femoral components on the range of motion of the hip at different head-neck ratios. J Bone Joint Surg Am 82(3):315–321 10724224 10.2106/00004623-200003000-00003

[9] Dargel J, Oppermann J, Bruggemann GP, Eysel P (2014) Dislocation following total hip replacement. Dtsch Arztebl Int 111(51–52):884–890 25597367 10.3238/arztebl.2014.0884 PMC4298240

[10] DiGioia AM, Jaramaz B, Blackwell M et al (1998) The Otto Aufranc Award. Image guided navigation system to measure intraoperatively acetabular implant alignment. Clin Orthop Relat Res. 10.1097/00003086-199810000-00003 9917587 10.1097/00003086-199810000-00003

[11] Emara AK, Zhou G, Klika AK et al (2021) Is there increased value in robotic arm-assisted total hip arthroplasty? A nationwide outcomes, trends, and projections analysis of 4,699,894 cases. Bone Joint J 103–B(9):1488–1496 34465149 10.1302/0301-620X.103B9.BJJ-2020-2411.R1

[12] Fernandez MA, Achten J, Parsons N et al (2022) Cemented or uncemented hemiarthroplasty for intracapsular hip fracture. N Engl J Med 386(6):521–530 35139272 10.1056/NEJMoa2108337

[13] Fessy MH, Putman S, Viste A et al (2017) What are the risk factors for dislocation in primary total hip arthroplasty? A multicenter case-control study of 128 unstable and 438 stable hips. Orthop Traumatol Surg Res 103(5):663–668 28629944 10.1016/j.otsr.2017.05.014

[14] Gausden EB, Popper JE, Sculco PK, Rush B (2020) Computerized navigation for total hip arthroplasty is associated with lower complications and ninety-day readmissions: a nationwide linked analysis. Int Orthop 44(3):471–476 31919568 10.1007/s00264-019-04475-y

[15] Gwam CU, Mistry JB, Mohamed NS et al (2017) Current epidemiology of revision total hip arthroplasty in the United States: national inpatient sample 2009 to 2013. J Arthroplasty 32(7):2088–2092 28336249 10.1016/j.arth.2017.02.046

[16] Han PF, Chen CL, Zhang ZL et al (2019) Robotics-assisted versus conventional manual approaches for total hip arthroplasty: a systematic review and meta-analysis of comparative studies. Int J Med Robot 15(3):e1990 30746868 10.1002/rcs.1990 PMC6594016

[17] Howell CC, Witvoet S, Scholl L, Coppolecchia A, Bhowmik-Stoker M, Chen AF (2025) Postoperative complications and readmission rates in robotic-assisted versus manual total knee arthroplasty: Large, propensity score-matched patient cohorts. J Am Acad Orthop Surg 33(2):83–91 39029449 10.5435/JAAOS-D-23-01117

[18] Inaba Y, Kobayashi N, Ike H, Kubota S, Saito T (2016) The current status and future prospects of computer-assisted hip surgery. J Orthop Sci 21(2):107–115 26850921 10.1016/j.jos.2015.10.023

[19] Jayaram RH, Gillinov SM, Caruana DL et al (2023) Total hip arthroplasty imageless navigation does not reduce 90-day adverse events or five-year revisions in a large National cohort. J Arthroplasty 38(5):862–867 36529197 10.1016/j.arth.2022.12.012

[20] Kamara E, Berliner ZP, Hepinstall MS, Cooper HJ (2017) Pin site complications associated with computer-assisted navigation in hip and knee arthroplasty. J Arthroplasty 32(9):2842–2846 28522245 10.1016/j.arth.2017.03.073

[21] Kennedy JG, Rogers WB, Soffe KE, Sullivan RJ, Griffen DG, Sheehan LJ (1998) Effect of acetabular component orientation on recurrent dislocation, pelvic osteolysis, polyethylene wear, and component migration. J Arthroplasty 13(5):530–534 9726318 10.1016/s0883-5403(98)90052-3

[22] Kirchner GJ, Lieber AM, Haislup B, Kerbel YE, Moretti VM (2021) The cost of robot-assisted total hip arthroplasty: comparing safety and hospital charges to conventional total hip arthroplasty. J Am Acad Orthop Surg 29(14):609–615 32991384 10.5435/JAAOS-D-20-00715

[23] Korber S, Antonios JK, Sivasundaram L et al (2021) Utilization of technology-assisted total hip arthroplasty in the United States from 2005 to 2018. Arthroplasty Today 12:36–44 34761092 10.1016/j.artd.2021.08.020 PMC8567325

[24] Krenzel BA, Berend ME, Malinzak RA et al (2010) High preoperative range of motion is a significant risk factor for dislocation in primary total hip arthroplasty. J Arthroplasty 25(6 Suppl):31–35 20541892 10.1016/j.arth.2010.04.007

[25] Kunze KN, Bovonratwet P, Polce EM, Paul K, Sculco PK (2022) Comparison of surgical time, short-term adverse events, and implant placement accuracy between manual, robotic-assisted, and computer-navigated total hip arthroplasty: a network meta-analysis of randomized controlled trials. J Am Acad Orthop Surg Glob Res Rev. 10.5435/jaaosglobal-d-21-00200 35472191 10.5435/JAAOSGlobal-D-21-00200 PMC10566925

[26] Learmonth ID, Young C, Rorabeck C (2007) The operation of the century: total hip replacement. Lancet 370(9597):1508–1519 17964352 10.1016/S0140-6736(07)60457-7

[27] Lin FF, Chen YF, Chen B, Lin CH, Zheng K (2019) Cemented versus uncemented hemiarthroplasty for displaced femoral neck fractures: a meta-analysis of randomized controlled trails. Medicine (Baltimore) 98(8):e14634 30813202 10.1097/MD.0000000000014634 PMC6407990

[28] Malik A, Maheshwari A, Dorr LD (2007) Impingement with total hip replacement. J Bone Joint Surg Am 89(8):1832–1842 17671025 10.2106/JBJS.F.01313

[29] Miki H, Yamanashi W, Nishii T, Sato Y, Yoshikawa H, Sugano N (2007) Anatomic hip range of motion after implantation during total hip arthroplasty as measured by a navigation system. J Arthroplasty 22(7):946–952 17920464 10.1016/j.arth.2007.02.004

[30] Montgomery BK, Bala A, Huddleston JI III, Goodman SB, Maloney WJ, Amanatullah DF (2019) Computer navigation vs conventional total hip arthroplasty: a medicare database analysis. J Arthroplasty 34(9):1994–1998 31176561 10.1016/j.arth.2019.04.063

[31] Mori Y, Tarasawa K, Tanaka H et al (2024) Does total hip arthroplasty in elderly patients with femoral neck fractures reduce complications? A Japanese DPC study. J Orthop Sci 38955576 10.1016/j.jos.2024.06.011

[32] Mori Y, Tarasawa K, Tanaka H et al (2024) Surgery on admission and following day reduces hip fracture complications: a Japanese DPC study. J Bone Miner Metab 42(5):608–615 38987506 10.1007/s00774-024-01534-2 PMC11455814

[33] Moskal JT, Capps SG, Scanelli JA (2013) Improving the accuracy of acetabular component orientation: avoiding malpositioning: AAOS exhibit selection. J Bone Joint Surg Am 95(11):e761–e710 23780546 10.2106/JBJS.L.00685

[34] Ogawa H, Hasegawa S, Tsukada S, Matsubara M (2018) A pilot study of augmented reality technology applied to the acetabular cup placement during total hip arthroplasty. J Arthroplasty 33(6):1833–1837 29502961 10.1016/j.arth.2018.01.067

[35] Ogawa H, Kurosaka K, Sato A, Hirasawa N, Matsubara M, Tsukada S (2020) Does an augmented reality-based portable navigation system improve the accuracy of acetabular component orientation during THA? A randomized controlled trial. Clin Orthop Relat Res 478(5):935–943 31834164 10.1097/CORR.0000000000001083 PMC7170692

[36] Parratte S, Argenson JN, Flecher X, Aubaniac JM (2007) [Computer-assisted surgery for acetabular cup positioning in total hip arthroplasty: comparative prospective randomized study]. Rev Chir Orthop Reparatrice Appar Mot 93(3):238–246 17534206 10.1016/s0035-1040(07)90245-7

[37] Piple AS, Wang JC, Hill W et al (2024) Postoperative outcomes and trends in computer-navigated and robotic-assisted total hip arthroplasty. Hip Int 34(5):569–577 39114946 10.1177/11207000241264256

[38] Remily EA, Nabet A, Sax OC, Douglas SJ, Pervaiz SS, Delanois RE (2021) Impact of robotic assisted surgery on outcomes in total hip arthroplasty. Arthroplasty Today 9:46–49 33997208 10.1016/j.artd.2021.04.003 PMC8105177

[39] Rowan FE, Benjamin B, Pietrak JR, Haddad FS (2018) Prevention of dislocation after total hip arthroplasty. J Arthroplasty 33(5):1316–1324 29525344 10.1016/j.arth.2018.01.047

[40] Shaw JH, Rahman TM, Wesemann LD, K GL-R CZJ, Davis JJ (2022) Comparison of postoperative instability and acetabular cup positioning in Robotic-Assisted versus traditional total hip arthroplasty. J Arthroplasty 37(8S):S881–S889 35143923 10.1016/j.arth.2022.02.002

[41] Simcox T, Singh V, Oakley CT, Koenig JA, Schwarzkopf R, Rozell JC (2022) Comparison of utilization and short-term complications between technology-assisted and conventional total hip arthroplasty. J Am Acad Orthop Surg 30(8):e673–e682 35139053 10.5435/JAAOS-D-21-00698

[42] Sloan M, Premkumar A, Sheth NP (2018) Projected volume of primary total joint arthroplasty in the U.S., 2014 to 2030. J Bone Joint Surg Am 100(17):1455–1460 30180053 10.2106/JBJS.17.01617

[43] Sugano N (2013) Computer-assisted orthopaedic surgery and robotic surgery in total hip arthroplasty. Clin Orthop Surg 5(1):1–9 23467021 10.4055/cios.2013.5.1.1 PMC3582865

[44] Tanaka H, Tarasawa K, Mori Y, Fushimi K, Fujimori K, Aizawa T (2024) Surgery within two days of admission reduces complications and mortality of patients with trochanteric femur fractures: a Japanese DPC study. Tohoku J Exp Med. 10.1620/tjem.2024.j093 39261079 10.1620/tjem.2024.J093

[45] Tanaka H, Tarasawa K, Mori Y et al (2025) Does osteonecrosis of the femoral head increase early complication rates after total hip arthroplasty? A Japanese nationwide medical claims database study. J Arthroplasty. 10.1016/j.arth.2025.01.029 39855403 10.1016/j.arth.2025.01.029

[46] Tomioka S, Rosenberg M, Fushimi K, Matsuda S (2020) An analysis of equity in treatment of hip fractures for older patients with dementia in acute care hospitals: observational study using nationwide hospital claims data in Japan. BMC Health Serv Res 20(1):830 32894116 10.1186/s12913-020-05690-9 PMC7487824

[47] World Health Organization (1992) International statistical classification of diseases TRI-World Health Organization

